# Mental health and disability among patients with chronic sciatica

**DOI:** 10.1192/j.eurpsy.2025.1898

**Published:** 2025-08-26

**Authors:** I. Sellami, A. Feki, A. Abbes, H. Bejaoui, Z. Gassara, M. Hajjaji, M. L. Masmoudi, K. Jmal Hammami, M. H. Kallel, H. Fourati, S. Baklouti

**Affiliations:** 1 Occupationnal medecine; 2 Rheumatology, CHU Hedi Chaker; 3 physical medicine and functional rehabilitation, CHU Habib Bourguiba, Sfax, Tunisia

## Abstract

**Introduction:**

Sciatica pain represents a typical symptom of spinal radicular syndromes. Disability due to this pain can affect mental health of patients.

**Objectives:**

Our study aims to assess the relationship between mental health and disability among patients with chronic sciatica.

**Methods:**

We conducted a descriptive, analytical and cross-sectional survey among patients suffering from documented common sciatic pain evolving for more than 3 months. We collected socio-professional data. We used the Hospital Anxiety and Depression Scale (HADS) and the Oswestry Disability Index (ODI).

**Results:**

Our study population was exclusively female, including 69 patients. The mean age of participants was 56.2 ±12.6 years. The most frequent etiology of sciatica pain was a herniated disc, followed by lumbar spinal stenosis and spondylolisthesis. The root path was L5 in 49 cases and S1 in 20 cases. The mean Oswestry score was 25 ± 4.1. The disability was moderate, severe and crippled respectively in 10.1%, 82.6% and 7.2% of patients. Regarding the patients’ anxiety levels, it was found that 76.8%, 20.3%, and 2.9% appeared to have mild, moderate, and severe anxiety, respectively. As for the depression levels of patients, 13 were mildly depressed (18.8%), 20 were moderately depressed (29%), and 36 were severely depressed (52.2%). We found that anxiety and depression were correlated with disability (p = <0.05, r = 0.2).

**Conclusions:**

Our findings highlight a correlation between altered mental health and disability among patients with chronic sciatica. It is crucial to screen psychiatric disorders among these patients in order to improve their well-being.

**Disclosure of Interest:**

None Declared

